# A Mixed‐Methods Evaluation of a Dietetic Outcome Measure Tool Pilot in a Community Eating Disorder Service

**DOI:** 10.1111/jhn.70346

**Published:** 2026-09-27

**Authors:** Steph Sloan, Jane Sadler, Sharleen Woods

**Affiliations:** ^1^ Teesside University Middlesbrough UK; ^2^ Lancashire and South Cumbria NHS Foundation Trust, Bamber Bridge UK

**Keywords:** dietetic outcomes, eating disorders, evaluation, outcome measures

## Abstract

**Background:**

Eating disorders (EDs) are associated with significant physical, psychological, and social impairments. Dietitians play a vital role in ED treatment, yet their impact is underreported.

**Aim:**

To evaluate the pilot implementation of a novel dietetic outcome measure tool within a community ED service, with a focus on feasibility and acceptability.

**Methods:**

A mixed‐methods evaluation was conducted over six months in 2024. Quantitative data were collected using a novel dietetic outcome measure tool being piloted within the service and analysed descriptively. Qualitative data were collected through semi‐structured interviews with dietitians piloting the tool and analysed using reflexive thematic analysis.

**Results:**

Quantitative outcome data from 102 patients were analysed to describe service level referral patterns, intervention focus, proposed outcomes, and perceived outcome achievement. Restrictive dietary patterns and malnutrition were identified as common dietetic concerns. Improving nutritional status and eating patterns were the most frequently identified dietetic outcomes. Outcome status was recorded for 54% of patients. Of those, twenty‐six (47%) had a recorded outcome as ‘met,’ 21 (38%) as ‘partially met,’ and 8 (15%) as “not met.” Qualitative findings are presented across 3 themes: (1) demonstrating professional value through outcome monitoring, (2) practical and organisational barriers to embedding outcome monitoring, and (3) balancing meaningful data collection with clinician burden.

**Conclusion:**

Participants were supportive of routine outcome monitoring in principle. However, the absence of clearly defined outcome indicators and associated data limited the tool's ability to meaningfully evaluate the intervention's effectiveness. Improved integration within existing electronic systems, combined with clearer outcome indicators and complementary qualitative approaches, may reduce clinician burden while enhancing the feasibility, acceptability, and value of routine outcome monitoring within ED dietetic services.

## Practitioner Points

- Outcome monitoring was supported by dietitians within the Community Eating Disorder Service but underutilised due to operational barriers, including time pressures and lack of tool integration within existing digital systems.
- ED care is multidisciplinary and nutritional needs are often multifactorial. This can make it difficult to isolate and measure the specific impact of dietetic care.
- While the outcome tool provided useful insights into dietetic practice within the service, its current format limited assessment of intervention effectiveness. Refining an outcome measure tool to include outcome indicators and qualitative feedback could strengthen the demonstration of impact and tool acceptability.

## 1 Introduction

Eating disorders (EDs) are psychological conditions that manifest as abnormal eating behaviours, preoccupation with food, and prominent body weight and shape concerns [1]. Untreated EDs cause significant harm to physical, psychological, and social well‐being. Among mental health conditions, EDs rank second only to substance‐use disorders in years of potential life lost (YPLL), with an average of 16.64 years lost [2]. YPLL is a widely used alternative measure for excess mortality, which represents the difference between the general population's lifespan and that of those living with a particular condition.

Suboptimal nutritional intake is well‐documented within ED populations [3] and physical complications seen in restrictive EDs are typically secondary to malnutrition [4]. Electrolyte abnormalities and associated medical complications are more common in EDs where purging symptoms present, such as Bulimia Nervosa [4]. Detriments to psychosocial functioning can be significant across all ED diagnoses, including binge eating disorder (BED) [5] and avoidant restrictive food intake disorder (ARFID) [6]. Participation and productivity within meaningful areas of life are significantly affected by EDs with the impact often extending to the lives of caregivers [7].

By nature, it is likely that most of not all patients within an ED service will present with some degree of nutritional risk and nutritional rehabilitation often a pertinent aim within treatment. In addition to nutritional imbalance and impairments in psychosocial functioning, individuals with EDs may be more vulnerable to incorrect or unhelpful dietary messages [8], making psychoeducation from a trustworthy source of knowledge an important component of treatment. Whilst research examining the quality of nutritional psychoeducation within ED teams is scarce, a small 2006 study identified dietitians to have the highest level of nutritional knowledge within an ED team where knowledge within some other professional groups was no different to laypersons [9].

The need for nutritional rehabilitation and access to reliable nutritional knowledge makes dietitians vital members of the ED multidisciplinary team (MDT) [10, 11, 12, 13]. Despite this, the dietetic role within ED care is often poorly defined, and with a lack of guidelines articulating the role, variations in practice exist [14]. Dietitians are presently under‐represented within ED services [15]. Compared to patients and carers, ED clinicians are less likely to endorse the need for dietetic involvement as a standard component of treatment [16]. Whilst evidence examining the impact of dietetic care within ED treatment is sparse, a consistent finding is that dietetic input improves body mass and nutritional intake [17].

It is therefore important for ED dietitians to enhance collection and reporting of outcomes to fully understand and articulate value within this practice area. To support this and as part of a wider quality improvement workstream to develop a dietetic pathway, dietitians within an all‐age community eating disorder service (CEDS) developed and piloted a dietetic outcome measure tool.

## 2 Aim

To evaluate the pilot implementation of a novel dietetic outcome measure tool within a CEDS, with a focus on feasibility and acceptability.

## 3 Objectives

1. To evaluate dietitians' experiences of piloting a novel dietetic outcome measure tool within a CEDS, including perceived barriers and facilitators to implementation.
2. To determine whether the pilot outcome measure tool supports meaningful collection and interpretation of outcome monitoring data capable of informing service development and improvement.
3. To generate recommendations to inform future refinement implementation of outcome monitoring processes within the CEDS dietetic service.

## 4 Methods

A mixed‐methods service evaluation was conducted to evaluate the pilot implementation of a novel dietetic outcome measure tool. This included exploration of barriers and facilitators to tool implementation (objective 1) alongside analysis of quantitative outcome tool data to determine whether the tool enabled meaningful and actionable service insights to be identified (objective 2). Results were then considered together to generate recommendations to inform refinement and implementation of the tool (objective 3).

A Masters (MSc) Dietetic student supported data collection and analysis as part of their studies. Trust approval for the service evaluation was obtained, and information governance advice was sought regarding transfer of anonymised service data to the student dietitian. University ethical approval was also obtained by the student (2024Jul19654 SADLER).

### 4.1 Setting

The evaluation formed part of a wider quality improvement workstream to develop a dietetic pathway within one CEDS in England. The all‐age CEDS provides assessment and treatment to those with EDs. Treatment options include evidence based 1:1 and group psychological interventions, physical health monitoring, and dietetic support. The dietetic service comprises seven registered dietitian posts (6.3 whole time equivalent), including one clinical specialist dietitian for each locality and two principle/consultant level dietitians with service wide clinical and strategic leadership responsibilities. Five of the seven dietetic posts were occupied at the time of the project. Dietitians' professional experience ranged from 8 to over 30 years, with all having at least 5 years of experience within ED services.

### 4.2 Outcome Tool Development

A novel outcome measure tool was developed by the dietitians in 2023 ahead of the pilot. Development was informed by professional body guidance relating to dietetic outcome measurement and standardised terminology [18, 19], alongside iterative team discussion to identify which professionally recognised dietetic outcomes were most relevant to community ED care and therefore inclusion within the tool. For example, those related to parenteral nutrition were unlikely to be relevant therefore were not included in the tool. Novel tool development aimed to align practices with professional body guidance and standardised dietetic terminology whilst maintaining clinical relevance and contextualisation to the service, an important consideration as clinician engagement with outcome monitoring is influenced by perceived relevance [20].

Drop‐down options were utilised where possible to aid tool completion and data interpretation. Data to be collected within the tool included referral information (referrer, referral criteria, date of referral, and risk rating), age, date of first and final contact, identified dietetic need, proposed dietetic outcome, outcome tracking status (whether outcome was met, end of episode outcome), and number of sessions provided during episode of care.

### 4.3 Quantitative Data

The dietitians utilised the tool for a 6‐month pilot period (January–June 2024), each populating an individual Microsoft Excel tool stored within a secure, password protected online server. All patient data used to populate the tool had been collected as part of routine dietetic care. All collected quantitative outcome data were then collated into one excel sheet by the principle investigator, anonymised and securely transferred to the student researcher for analysis using descriptive statistics, including counts, percentages, and measures of central tendency.

Whilst dietitians were asked to populate the tool for all new episodes of care commenced during the 6‐month pilot period, implementation appeared to vary with some also populating for existing episodes of patient care. This is explored further within the discussion and gives context to some practice variations, i.e., number of sessions provided.

### 4.4 Qualitative Data

Qualitative data were collected by the student researcher through a series of semi‐structured interviews conducted with the dietitians over a 4‐week period following the 6‐month tool pilot phase. A semi‐structured interview guide was developed informed by evaluation aims, relevant literature and supervisor feedback. Interviews were conducted, recorded, and transcribed via Microsoft Teams. The student researcher undertook the evaluation as part of a supervised research project during the final semester of a pre‐registration dietetic MSc programme. They had completed a mental health and ED clinical placement elsewhere in the country, providing some contextual understanding of the specialism but had not worked within the participating service. The lead author supervised the project as the students university course tutor and was an experienced ED dietitian who had undertaken casual clinical work within the participating service to maintain their clinical practice. Consequently, the lead author was known to participants. These differing perspectives and experiences were acknowledged throughout the analytic process through ongoing reflexive discussion, supported by a reflective journal maintained by the student researcher during data collection and analysis.

Following data cleaning and anonymisation, qualitative data were analysed using reflexive thematic analysis following Braun and Clarke's six phase approach [21 p. 35–36]. The student researcher first familiarised themselves with the dataset through repeated reading of transcripts before generating inductive codes. Codes were then iteratively reviewed, grouped, and refined to construct themes capturing dietitians' experiences of piloting the outcome tool. Themes were subsequently refined through supervisory discussion. Whilst code checking was not undertaken, consistent with a reflexive paradigm [21 p. 238–239], the student researcher received regular supervisory support throughout the analytic process. Rather than verifying coding decisions, supervision provided a space for reflexive dialogue, critical questioning, and exploration of alternative interpretations. This was particularly important given the researcher's novice status and supported the development of a rigorous and reflexive analysis. Quantitative and qualitative data were analysed and reported separately, with qualitative findings largely addressing evaluation objective 1 and quantitative data objective 2. Findings from both components were then considered together through an integrated interpretation process, enabling a more comprehensive understanding of the pilot by combining service‐level outcome data with clinicians' experiences of implementation. This integration supported the development of overall conclusions regarding the pilot's feasibility and acceptability, alongside informing practice recommendations (objective 3).

## 5 Results

Populated outcome measure tools were submitted by all five eligible CEDS dietitians, collectively representing 102 unique patients. Quantitative findings present service‐level data relating to referral patterns, intervention focus, proposed outcomes, and perceived outcome achievement. Four of the five dietitians participated in a semi‐structured interview. Qualitative findings describe dietitians' experiences of piloting the novel outcome tool, including perceived barriers and facilitators to implementation, across three themes: (1) demonstrating professional value through outcome monitoring, (2) practical and organisational barriers to embedding outcome monitoring, and (3) balancing meaningful data collection with clinician burden.

### 5.1 Quantitative Results

#### 5.1.1 Referrals

A referral source was recorded for all 102 patients. Most referrals to the CEDS dietitians originated from MDT meetings (*n* = 44, 43%), followed by case managers (*n* = 40, 39%). The remaining referrals (*n* = 18, 18%) came from alternative referral routes, which were not further described. Of the 5 CEDS dietitians, dietitian 1 was most likely to receive referrals via the MDT route (*n* = 8, 62%) whilst dietitian 5 received only 29% (*n* = 6) via the MDT. Dietitians 2, 3, and 4 had similar referral route patterns receiving 44% (*n* = 12), 48% (*n* = 13), and 42% (*n* = 5), respectively, via the MDT.

Sixteen patients (19%) had no referral reason documented within the outcome tool. Of those with a documented referral reason (*n* = 86), ‘weight faltering’ was most common (*n* = 28, 33%) followed by ‘atypical presentation, including neurodivergence’ (*n* = 13, 15%) and ‘high risk re‐feeding syndrome’ (*n* = 10, 12%). Less common referral reasons included ‘nutrition related comorbidity’ (*n* = 5, 6%), ‘purging with electrolyte abnormality’ (*n* = 5, 6%), ‘extended assessment’ (*n* = 3, 3%), ‘BED’ (*n* = 2, 2%), ‘lack of parental confidence’ (*n* = 1, 1%), and ‘perinatal’ (*n* = 1, 1%). The remaining 17 patients with a recorded reason for referral were categorised as ‘other’ (20%). Whilst comparison of referral reasons between dietitians was not formally undertaken, some dietitians were more likely to select ‘other,’ e.g., dietitian 4 selected this for 42% (*n* = 5) of their referrals whilst all instances where no reason for referral was recorded were observed within dietitian 3's populated outcome measure tool. Figure 1 details the pooled number of referrals received for each referral reason.

**Figure 1:**
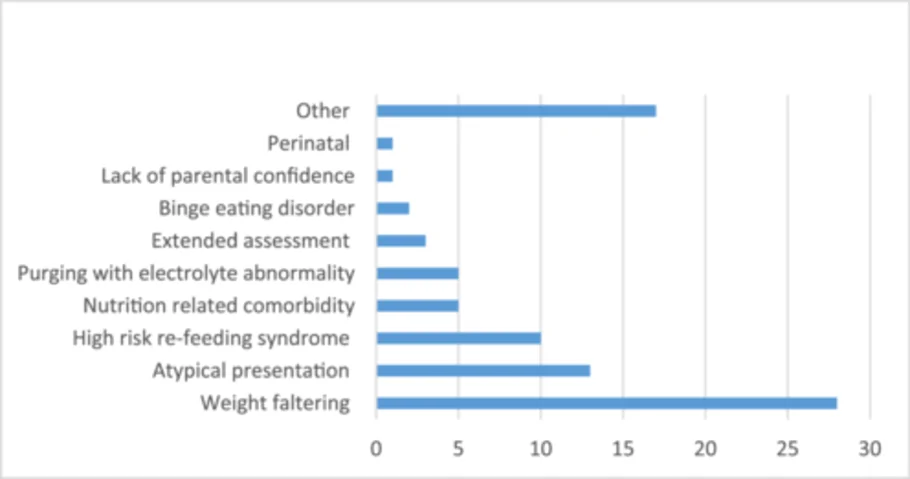
Number of referrals received for each referral reason.

Mean patient age at time of referral was 23 years old with a range of 9–63 years. Thirty‐one referrals (30%) were for children and young people (CYP) (< 18 years old). Dietitian 4 had the largest proportion of CYP referrals at 67% (*n* = 8) whilst dietitians 1 and 3 had only 15% (*n* = 2) and 15% (*n* = 4) of their referrals for CYP, respectively.

### 5.2 Dietetic Intervention

Most patients had a documented dietetic need within the outcome measure tool with only 6 (6%) missing. The most frequently selected dietetic need was ‘restrictive dietary pattern’ (*n* = 31, 32%) followed by ‘malnutrition’ (*n* = 31, 32%). Others included ‘high risk of re‐feeding syndrome’ (*n* = 7, 7%), ‘altered biochemistry’ (*n* = 6, 6%), ‘incomplete knowledge’ (*n* = 6, 6%), ‘energy imbalance’ (*n* = 6, 6%), ‘limited foods’ (*n* = 4, 4%), ‘faltering growth’ (*n* = 3, 3%), and '‘macronutrient imbalance'' (*n* = 1, 1%). Two patients were identified as having no dietetic need following dietetic assessment and 3 as ‘other.’

Most patients had a documented proposed outcome within the tool with only 8 (8%) missing. Of those with a proposed outcome (*n* = 94), 38 (40%) were ‘improve nutritional status,’ 19 (20%) ‘improve eating pattern,’ 11 (12%) ‘prevent inpatient admission,’ 4 (4%) ‘quality of life,’ 3 (3%) ‘parent/carer empowerment,’ 3 (3%) ‘improve food variety,’ 3 (3%) ‘maintain BMI/centiles,’ 2 (2%) ‘minimise losses,’ 2 (2%) ‘improve growth and development,’ and the remaining 10 (11%) ‘other.’

All dietitians were most likely to select ‘improve nutritional status’ as their primary proposed dietetic outcome. Similarly, all dietitians utilised the ‘improve eating pattern’ and ‘minimise losses’ proposed outcome within the tool. All other proposed outcomes were unused by at least 1 dietitian. Figure 2 displays proposed dietetic outcomes most selected by each dietitian as a percentage of their total recorded cases.

**Figure 2:**
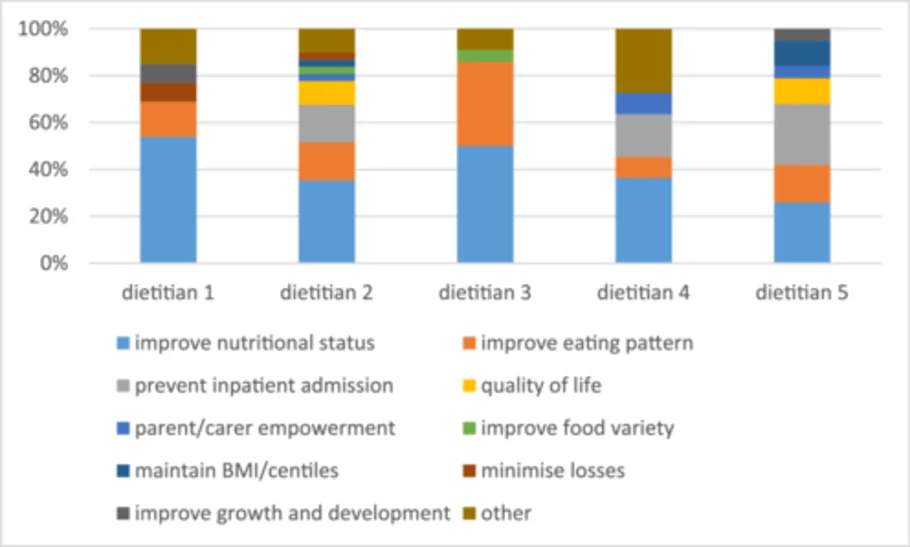
Proposed dietetic outcomes for each dietitian as a percentage of their total recorded cases.

Outcome status was recorded for 55 patients (54%). Of those, twenty‐six (47%) had a recorded outcome as ‘met,’ 21 (38%) as ‘partially met,’ and 8 (15%) as ‘not met.’ The remaining 47 patients (46%) did not have this information recorded within the outcome tool.

Information regarding the number of sessions provided within dietetic episode of care was recorded for most patients (*n *= 67, 66%). Of those patients with this information recorded, 19 (28%) were seen for 1–4 sessions, 8 (12%) for 5–10 sessions, 6 (9%) for 11–20 sessions, 1 (1%) for 21–30 sessions, 15 (22%) for 31–50 sessions, and 4 (6%) for > 50 sessions. The number of sessions provided to patients varied between dietitians whereby 100% recorded by dietitian 4 (*n* = 12) were seen for 1–4 sessions whilst dietitian 5 saw only 24% (*n* = 5) of referrals for 1–4 sessions. Only dietitians 2 and 3 provided > 50 sessions to patients. Figure 3 displays the number of dietetic sessions provided to patients by each dietitian as a percentage of their total recorded cases.

**Figure 3:**
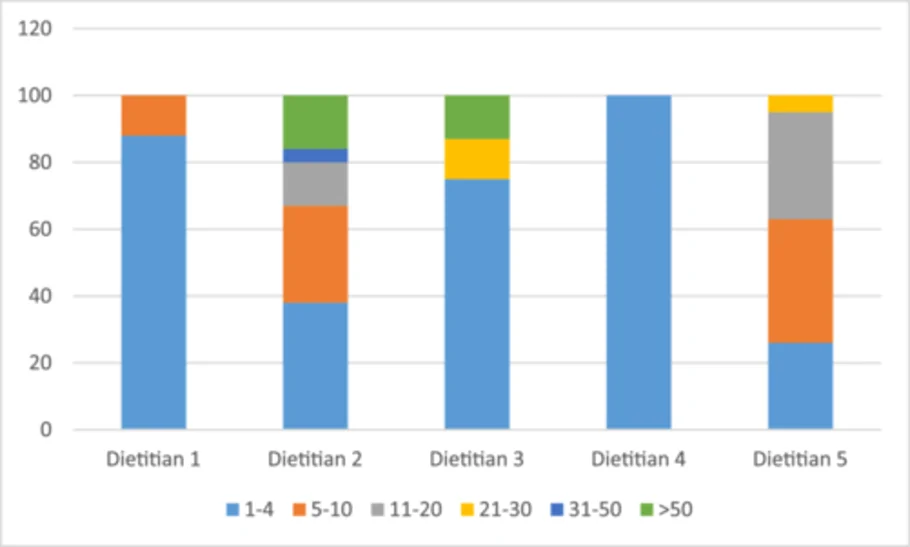
Number of dietetic sessions provided to patients with an episode of care, presented per dietitian as a percentage of their total recorded cases.

### 5.3 Qualitative Results

Qualitative semi‐structured interview results are presented via 3 themes.

Theme 1: Demonstrating professional value through outcome monitoring

Whilst participants recognised the importance of using outcome measures, they acknowledged that as a profession, demonstrating impact is rarely prioritised.

‘I think it's important moving forward as dietitians that we do, you know, show what we're doing and our value, but more even in terms of service development, in terms of you know identifying whether we need to have more dietitians.’ Participant 2.

‘We've been saying for years we need outcome measures, so let's start.’ Participant 4.

Participants acknowledged challenges measuring impact particularly within ED, due to care being multidisciplinary with more than one clinician involved.

‘Yeah, I think as a profession, we're not very good at collecting outcomes. And I'd say that's even more so within the ED field, because there's so many other practitioners involved in an MDT. Then how do you measure us individually as a profession that can be really hard.’ Participant 1.

Theme 2: Practical and organisational barriers to embedding outcome monitoring

Participants described difficulties integrating outcome monitoring into routine clinical practice due to competing demands, limited time, and challenges associated with the functionality and lack of integration with existing recording systems. All participants populated the tool retrospectively and felt that adapting to a new way of working in recording outcomes data was time consuming.

‘limited time to stop and do that as well as patient electronic notes and everything else that's happening in the day’ Participant 3.

‘it's an easy thing to do in a way, but then because you've got so much to do, it kind of just naturally fell at the bottom of the priorities list’ Participant 2.

Participants encountered some functionality issues with the tool, such as finding the drop‐down options limiting.

‘Like there was very specific drop downs and I had to just do the best fit even though it wasn't exactly what I was doing.’ Participant 1.

Participants often related this to the ED practice area, noting that dietetic need is rarely driven by a single identifiable cause.

‘But within eating disorders, it's quite, you know, like they don't get malnourished just because, they get malnourished because of a restrictive diet pattern.’ Participant 2.

Another perceived barrier was that particularly when supporting neurodivergent patients, forming a therapeutic relationship was an important component of care that was not easily captured within the tool.

‘It was very difficult to measure the outcome because most of my caseload are neurodivergent. So a lot of the time it's building an initial relationship before you move into a piece of work.’ Participant 1.

Participants noted that the tools placement in a separate Excel document, rather than being integrated into the existing electronic system, limited its functionality and ease of use.

‘I think when we're trying to fill in like our patient electronic system, and then remember about a spreadsheet as well, when things are not just together in one place then it's difficult to kind of do everything that we have to remember to do.’ Participant 1.

Theme 3: Balancing meaningful data collection with clinician burden

Participants were supportive of further developing an outcome monitoring process within the service and suggested refinements, including to reduce completion burden and maximise meaningful value of the process. Improvements included refining categories within the tool, populating age in years only (as opposed to years, months) and removing date of referral. Participants also suggested the inclusion of patient and MDT qualitative feedback as part of outcome monitoring. One participant noted the need for a consistent approach to collecting outcomes to obtain data that can derive meaningful conclusions.

‘So actually being quite isolated has then meant that we're probably doing it quite differently. It's only as useful as how you know if you're comparing everyone together, you have to be completing it in the same way.’ Participant 2.

## 6 Discussion

To our knowledge, limited literature has explored the use of dietetic outcome measure tools within ED. This evaluation therefore contributes to an underdeveloped area of practice by piloting routine outcome monitoring within a specialist ED service. Consistent with existing literature [20, 22, 23], dietitians perceived outcome monitoring as an important means of evidencing the contribution and value of dietetic practice, reflected in theme 1 *(Demonstrating professional value through outcome monitoring)* and supported by the willingness of all 5 eligible dietitians to provide outcome data during the pilot. However, several barriers were identified *(theme 2: Practical and organisational barriers to embedding outcome monitoring),* including concerns of overly reductive or standardised approaches to capturing multifaceted changes. These concerns were reinforced by data omissions within populated outcome tools. Participants also shared several practical recommendations to support meaningful outcome monitoring whilst managing clinician burden *(theme 3: Balancing meaningful data collection with clinician burden)*. Drawing on findings, we propose several recommendations for dietetic outcome monitoring within ED and present a provisional dietetic outcome and outcome indicator framework (Appendix S1) that would benefit from further co‐design and validation.

Health outcomes within dietetic practice are often influenced by multiple factors, making it difficult to establish a direct cause and effect relationship between dietetic intervention and patient outcomes [24]. This challenge may be particularly pronounced within ED care, which is multidisciplinary [12, 25], and multiple interventions may collectively contribute to recovery, including diet and nutrition related outcomes [26]. The combination of interventions offered will vary according to an individual's needs, treatment priorities, and local service provision. For example, an individual with a restrictive ED may receive a combination of psychological therapy, dietetic intervention, physical health monitoring, medication management, occupational therapy, and mealtime support, all of which may contribute to weight restoration and improved health. This complexity was reflected in the present evaluation where participants identified difficulties isolating the specific contribution of dietetic intervention to observed outcomes as a barrier to embedding outcome monitoring within practice (Theme 1). Whilst acknowledging as an expected limitation of profession or intervention specific outcome monitoring, evaluation findings may suggest value in placing greater emphasis on outcomes more directly and specifically aligned to dietetic intervention. On outcome tool completion, participants most commonly selected ‘improving nutritional status’ as the primary dietetic goal. Although this can represent an important overarching aim of dietetic care, its broad nature may contribute to difficulties attributing change to dietetic intervention alone. Supporting dietitians to define more specific nutrition‐related goals, such as improvements in macronutrient, micronutrient and/or energy intake, diet quality, or nutritional knowledge, may enhance the sensitivity of outcome monitoring to dietetic intervention, while broader outcomes, including weight restoration and psychosocial functioning, continue to be monitored as indicators of overall multidisciplinary treatment progress.

International standards recommend that ED dietetic outcome monitoring incorporates both quantitative and qualitative approaches [26], reflecting the holistic nature that ED dietitians wish to practice by, extending beyond weight restoration to encompass an individual's relationship with food and broader recovery [14]. Evaluation findings suggest that the piloted outcome tool did not fully capture this preferred holistic approach to practice. Participants described structured drop‐down options within the outcome tool as restrictive, reporting that the multifaceted nature of ED presentations and dietetic care could not always be adequately represented through predefined categories (Theme 2), further reflected by frequent use of the ‘other’ response option within the tool. Participants also recommended incorporating qualitative MDT perspectives alongside quantitative outcome data to better capture meaningful outcomes in care (Theme 3). Embedding qualitative approaches within outcome monitoring may therefore help capture profession‐specific patient experiences, alongside the relational, collaborative, and contextual aspects of dietetic care that are less easily quantified. Such adaptations may enhance the perceived value of outcome monitoring for ED dietitians, important given that perceived relevance and value are facilitators to successful outcome monitoring implementation [20].

Beyond ensuring that outcome monitoring captures meaningful aspects of dietetic care, successful implementation also depends on its usability within routine clinical practice. Consistent with existing literature [20, 22, 23], clinician burden was identified as a barrier, with dietitians perceiving outcome monitoring as an additional responsibility within an already pressured role. However, findings from both the semi‐structured interviews (Theme 2) and omissions within completed outcome tools suggest that tool functionality represented an important and potentially modifiable contributor to this burden. Optimising system functionality is an important facilitator to effective outcome monitoring [20, 22]; therefore better integration with existing electronic patient record systems, rather than requiring completion through a separate platform, may support implementation.

Outcome monitoring should be both simple and comprehensive [27]. However, demonstrating short‐term indicators of impact can be challenging in dietetics where intended outcomes may take time to emerge [22], particularly within ED care where recovery is often neither rapid nor linear. This is reflected within theme 2 where participants highlighted the therapeutic value of rapport building despite difficulties quantifying through routine outcome monitoring. Particular attention was drawn to early stages of intervention when establishing therapeutic rapport and trust can be the primary focus of dietetic care, and when working with neurodivergent individuals. Individuals with EDs highly value therapeutic relationships with dietitians [28] and neurodivergence and comorbidities are common within ED populations [29]. Consistent with this, ‘atypical presentation, including neurodivergence,’ was the second most frequently selected referral reason within the present evaluation. Flexible outcome monitoring approaches that acknowledge the importance of therapeutic alliance, even if not readily quantified, may therefore be needed if to reflect clinical realities. Similar concerns regarding overly standardised outcome monitoring have also been identified across wider allied health settings, where failure to capture clinical complexity is recognised as a barrier to implementation [20].

Whilst lack of knowledge or confidence in tool completion was not explicitly identified within interviews, it may be indirectly reflected through data omissions and apparent variation in tool use between dietitians. For example, dietitian 4 reported all patients receiving 1–4 sessions, whereas dietitians 2 and 3 reported some patients receiving more than 50 sessions. This may reflect differences in whether the tool was completed only for new referrals received during the pilot or for existing caseloads, suggesting variation in interpretation of the pilot guidance. Dietitian 4 also reported a larger proportion of CYP referrals and made greatest use of the ‘other’ referral category, which may indicate that existing referral categories are less applicable to CYP populations. In contrast, the ‘dietetic need’ and ‘proposed outcome’ sections were completed for most patients, suggesting these sections were either more intuitive to complete or required less refinement. Regardless, qualitative findings highlighted the importance of a consistent approach to outcome monitoring if meaningful conclusions are to be drawn (theme 3).

In addition to understanding dietitians' experiences piloting the tool, the evaluation also sought to determine whether the novel outcome tool enabled meaningful data collection and actionable insight generation. The British Dietetic Association defines the purpose of outcome measures as assessing the validity, reliability, and responsiveness of interventions [18]. Outcome monitoring may benefit clinicians, e.g., supporting decision making; patients, e.g., through providing reassurance regarding effectiveness of care; and commissioners, e.g., through demonstrating value. Within ED care, where the dietetic role is poorly defined [14] and under‐represented [15], monitoring impact is crucial to advance our practice and articulate value.

The pilot generated several useful insights into dietetic practice within the service. Dietetic referral process appeared to vary between dietitians, suggesting further exploration may be beneficial should the service wish to standardise referral pathways. Referral reasons infrequently selected, including ‘lack of parental confidence,’ ‘perinatal,’ and ‘BED’ may warrant service discussion to establish whether dietetic needs within these presentations are being consistently recognised and referred appropriately. Variation in the reported number of dietetic sessions offered may also warrant exploration, although, as discussed above, may partly reflect differences in tool completion rather than true variation in clinical practice.

The tool required dietitians to identify a proposed dietetic outcome and subsequently record whether this was ‘met,’ ‘partially met,’ or ‘not met.’ Although proposed outcomes were recorded for most patients, outcome achievement status was only documented for around half. This may reflect a limitation of the six‐month pilot period, as some dietetic interventions were likely to remain ongoing when data collection concluded. Consequently, outcome data may have been entered before interventions were complete, limiting interpretation of outcome achievement. Where outcome achievement status was recorded, approximately half were reported as ‘met’ and a further third as ‘partially met,’ suggesting that most interventions provided at least some perceived benefit. However, as no outcome indicator data (e.g., energy intake, BMI, and %weight change) were recorded, with reliance on dietitians' subjective assessment of outcome achievement, the ability to draw credible conclusions regarding effectiveness of dietetic intervention is limited.

## 7 Limitations

This evaluation was undertaken within a single specialist CEDS and was designed to inform local service development. The participating dietitians were an experienced specialist workforce, and findings should therefore be interpreted within this context. Consideration should be given to differences in service models and workforce composition if considering any transferability to other settings.

Although four of the five eligible dietitians participated in the interviews, participation was voluntary and some selection bias is possible. In addition, as participants were evaluating an outcome tool developed within their own service, responses may have been influenced by social desirability bias.

The evaluation relied on routinely collected clinical information, which was incomplete for some variables, often entered retrospectively and recorded inconsistently between dietitians. For example, some dietitians appeared to record outcome data for new referrals only, whereas others appeared to include their existing caseload. Consequently, data completeness and consistency varied across records. Furthermore, the six‐month evaluation period provided only a short‐term assessment of implementation and feasibility.

The most significant limitation of the piloted outcome tool itself was the omission of objective or standardised pre‐intervention and post‐intervention outcome indicator data, limiting the ability to draw meaningful conclusions regarding the effectiveness of intervention.

Finally, this evaluation focused on the feasibility and acceptability of implementing a locally developed outcome tool within routine clinical practice and formal psychometric evaluation was beyond the scope of the project. Future work could build on this pilot by involving patients in the co‐design and refinement of the tool, followed by formal psychometric evaluation, including assessment of validity, internal consistency, and responsiveness, alongside evaluation of feasibility and acceptability across a broader range of clinical settings.

## 8 Conclusion

This service evaluation explored the pilot implementation of a novel dietetic outcome measure tool within a CEDS as part of a wider quality improvement workstream developing a dietetic pathway. Dietitians were supportive of routine outcome monitoring in principle, recognising its potential to demonstrate professional value. However, the evaluation also identified important barriers to implementation, including clinician burden, time constraints, limited digital functionality, restrictive outcome categories, and difficulties attributing change specifically to dietetic intervention within multidisciplinary ED care. While the tool provided useful insights into referral patterns, intervention focus, and perceived outcomes within the service, the absence of clearly defined outcome indicators and associated outcome data limited its ability to draw meaningful conclusions regarding intervention effectiveness. Findings suggest that whilst dietitians supported the principle of routine outcome monitoring, further refinement of the tool is needed before routine implementation. Priorities include improved integration within electronic systems, incorporation of meaningful outcome indicator data, and complementary qualitative approaches to enhance the feasibility, acceptability, and credibility of dietetic outcome monitoring within the CEDS.

Despite limitations, this evaluation provides valuable early insight into an understudied area of clinical practice. By exploring the implementation and acceptability of a locally developed outcome tool, it offers an important first step towards developing more robust approaches to evaluating the contribution and impact of dietetic care in ED treatment.

### 8.1 Recommendations

1. Outcome monitoring should be built into existing electronic systems to reduce completion burden.
2. The utilisation of an outcome monitoring tool or process must be standardised amongst dietitians within the team to ensure consistency and reliability.
3. Simplification of data entry with the removal of data deemed to be low value:
  a. Patient age in years and months
  b. Date referral received.
4. Inclusion of qualitative patient and MDT feedback to capture the broader impact of dietetic care.
5. Embed pre/post outcome indicator for each dietetic outcome measure to increase quality and credibility of conclusions drawn from outcome data. Aim to align with professional body standardised language terminology. See Appendix S1 for a provisional framework for dietetic outcomes and outcome indicators, which would benefit from further co‐design and validation.
6. Revisit referral criteria during pathway review, focusing on suitability for CYP.

## Author Contributions

**Steph Sloan:** led conceptualisation and design, contributed to data interpretation, and led the drafting and revision of the manuscript. **Jane Sadler:** contributed to the development of the study design, undertook data collection and interpretation and undertook revision of the manuscript. **Sharleen Woods:** contributed to data analysis and interpretation and assisted in manuscript preparation and revision.

## Funding

The authors have nothing to report.

## Ethics Statement

Obtained from Teesside University School of Health and Life Sciences AHP Research Ethics sub‐Committee. Approval number: 2024Jul19654 SADLER.

## Conflicts of Interest

The authors declare no conflicts of interest.

## Supporting information


**Appendix 1:** Provisional Dietetic Outcome and Outcome Indicator Framework.

## Data Availability

The data that support the findings of this study are available from the corresponding author upon reasonable request.
