# Supplementary material for: A Mixed‐Methods Evaluation of a Dietetic Outcome Measure Tool Pilot in a Community Eating Disorder Service
Source: J Hum Nutr Diet. 2026 Sep 27;39(5):e70346. doi: 10.1111/jhn.70346 (PMC13617232; doi:10.1111/jhn.70346)
Supplement: Supplementary file 1 [file JHN-39-0-s001.docx]

**Appendix 1**: Outcome indicators for eating disorder dietetic care

| **Outcome Category** | **Outcome** | **Outcome indicator** |
| --- | --- | --- |
| **Clinical** | Improve weight / BMI / BMI centile / %wfh / %mBMI | Weight (kg)  BMI (kg/m2)  BMI centile  %wfh / %mBMI |
|  | Maintain weight / BMI / BMI centile / %wfh / %mBMI | Weight (kg)  BMI (kg/m2)  BMI centile  %wfh / %mBMI |
|  | Improve nutritional status | MUAC  Hand grip strength  BMI (kg/m2)  BMI centile  %wfh / %mBMI |
|  | Maintain nutritional status | MUAC  Hand grip strength  BMI (kg/m2)  BMI centile  %wfh / %mBMI |
|  | Increase growth | Growth rate (centile deviation) |
|  | Minimise losses | Weight loss (kg)  Weight loss (%bw) |
|  | Reduce risk of complications | Target symptom frequency and intensity (e.g., number of purging episodes, compulsive exercise) |
|  | Reduce risk of complications: biochemical/haematological abnormality | Biochemical / haematological markers |
|  | Reduce risk of complications: bowels | Bristol stool chart  Stool frequency |
|  | Reduce risk of hospitalisation | Number of MEED red flags |
| **Dietary** | Improve restrictive eating behaviour | Number of eating occasions in the day |
|  | Improve variety in nutritional intake | Number of daily food group recommendations achieved  Food frequency questionnaire |
|  | Improve macronutrient intake | Carbohydrate intake (number of servings/d)  Carbohydrate intake (g/d)  Protein intake (number of servings/d)  Protein intake (g/d)  Fat intake (number of servings/d)  Fat intake (g/d)  Fibre intake (g/d) |
|  | Improve micronutrient intake | %requirement achieved |
|  | Improve energy intake | Energy intake (kcal/d)  Energy intake (%requirements achieved) |
|  | Reduce artificial nutrition | Kcals from artificial nutrition  %requirements from artificial nutrition |
|  | Improve hydration | Fluid intake (ml/d)  Fluid intake (%requirements achieved)  Urea and Electrolytes |
| **Behavioural** | Improve patient empowerment | Patient reported confidence scale  Patient reported motivation scale  Readiness to change ruler  Anorexia Nervosa Stages of Change Questionnaire  The Warwick-Edinburgh Mental Wellbeing Scales (WEMWBS) score |
|  | Improve knowledge | Observed achievement of goal |
|  | Parent / carer empowerment | Parent/carer reported confidence scale  Parent / carer reported motivation scale  Readiness to change ruler |
|  | Improve quality of life | The World Health Organization Quality of Life (WHOQOL-BREF) |
| **Psychological** | Eating disorder cognitions | Eating Disorder Examination Questionnaire (EDEQ)  Body Shape Questionnaire (BSQ) |
